# Uncomplicated *Plasmodium vivax* malaria: mapping the proteome from circulating platelets

**DOI:** 10.1186/s12014-021-09337-7

**Published:** 2022-01-05

**Authors:** Diana Fernández, Cesar Segura, Mònica Arman, Suzanne McGill, Richard Burchmore, Tatiana Lopera-Mesa

**Affiliations:** 1 Malaria Group, Facultad de Medicina, Universidad de Antioquia, Calle 62 # 52-59, Torre 1, Lab 610, Sede de Investigación Universitaria, Medellin, Colombia; 2 Centre for Atherothrombosis and Metabolic Disease, Hull York Medical School, Faculty of Health Sciences, University of Hull, Hull, UK; 3 Institute of Infection, Immunity and Inflammation and Glasgow Polyomics, College of Medical, Veterinary and Life Sciences, University of Glasgow, Glasgow, UK

**Keywords:** *Plasmodium vivax*, Thrombocytopenia, Platelets proteome

## Abstract

**Background:**

Thrombocytopenia is frequent in *Plasmodium vivax* malaria but the role of platelets in pathogenesis is unknown. Our study explores the platelet (PLT) proteome from uncomplicated *P. vivax* patients, to fingerprint molecular pathways related to platelet function. Plasma levels of Platelet factor 4 (PF4/CXCL4) and Von Willebrand factor (VWf), as well as in vitro PLTs—*P. vivax* infected erythrocytes (*Pv*-IEs) interactions were also evaluated to explore the PLT response and effect on parasite development.

**Methods:**

A cohort of 48 patients and 25 healthy controls were enrolled. PLTs were purified from 5 patients and 5 healthy controls for Liquid Chromatography–Mass spectrometry (LC–MS/MS) analysis. Plasma levels of PF4/CXCL4 and VWf were measured in all participants. Additionally, *P. vivax* isolates (n = 10) were co-cultured with PLTs to measure PLT activation by PF4/CXCL4 and *Pv-*IE schizonts formation by light microscopy.

**Results:**

The proteome from uncomplicated *P. vivax* patients showed 26 out of 215 proteins significantly decreased. PF4/CXCL4 was significantly decreased followed by other proteins involved in platelet activation, cytoskeletal remodeling, and endothelial adhesion, including glycoprotein V that was significantly decreased in thrombocytopenic patients. In contrast, acute phase proteins, including SERPINs and Amyloid Serum A1 were increased. High levels of VWf in plasma from patients suggested endothelial activation while PF4/CXCL4 plasma levels were similar between patients and controls. Interestingly, high levels of PF4/CXCL4 were released from PLTs—*Pv*-IEs co-cultures while *Pv*-IEs schizont formation was inhibited.

**Conclusions:**

The PLT proteome analyzed in this study suggests that PLTs actively respond to *P. vivax* infection. Altogether, our findings suggest important roles of PF4/CXCL4 during uncomplicated *P. vivax* infection through a possible intracellular localization. Our study shows that platelets are active responders to *P. vivax* infection, inhibiting intraerythrocytic parasite development. Future studies are needed to further investigate the molecular pathways of interaction between platelet proteins found in this study and host response, which could affect parasite control as well as disease progression.

**Supplementary Information:**

The online version contains supplementary material available at 10.1186/s12014-021-09337-7.

## Background

*Plasmodium vivax* infection is widespread outside Africa [1]. South America, and specifically Colombia, are considered low endemic malaria regions where *P. vivax* accounts for ~ 50% of malaria cases [2]. *P. vivax* malaria has been historically considered benign, mainly due to its limited replication rates in reticulocytes, that circulate at low proportion (1.5%) in blood [3]. However, it is well known that even at lower parasitemia rates, *P. vivax* infections can lead to hemostasis dysregulation [4].

Thrombocytopenia defined as a reduced blood platelet (PLT) count, is frequent in malaria cases, and it has been described in ~ 49% of *P. vivax* malaria patients in Colombia [5]. The causes of thrombocytopenia in *P. vivax* infection are unclear, but they have been related to increased PLT destruction (including the production of temporary anti-PLTs antibodies and destruction by macrophages) or higher activation/consumption [6–13], rather than decreased production. Despite the high frequency of thrombocytopenia, neither the role of PLTs in *P. vivax* malaria pathogenesis nor the consequences of interactions between platelets and *P. vivax* infected erythrocytes (Pv-IEs) are understood.

PLT structure, functions, and molecular activation pathways are better understood in the context of cardiovascular diseases [14]. An estimated ~ 5000 PLT proteins, ~ 13,600 protein–protein interactions, and 229 PLT kinases have been described (http://plateletweb.bioapps.biozentrum.uni-wuerzburg.de/plateletweb.php) based on several proteomic approaches [15, 16]. These studies have shed some light on the function of PLTs in hemostasis. The role of PTLs in immune response to infections has been also studied. Recently, the PLT proteome from patients with Dengue Virus, where thrombocytopenia is a hallmark, showed alterations in protein expression (e.g., PF4/CXCL4 and HLA class I proteins associated with antigen processing and presentation) [17].

PLTs have several functions including regulation of hemostasis, vascular integrity, inflammation and immune response [18]. In *P. falciparum* malaria, the role of PLTs has been extensively studied [19, 20], including their participation on IEs sequestration on the microvasculature by bridging IEs to endothelial cells (ECs) [7, 21]. Also, in vitro studies have shown direct binding between PLTs and *P. falciparum* IEs (*Pf*-IEs) forming aggregates, a phenotype known as PLT-mediated clumping [11]. Moreover, PLT activation is believed to contribute to cerebral vascular damage by inducing expression of EC adhesion molecules, leading to more PLT-endothelial interactions and cerebral vascular localization of *Pf*-IEs [21, 22]. In contrast, in *P. vivax* malaria, sequestration of *Pv*-IE is rare, but the imbalanced pro-inflammatory response is high compared with *P. falciparum* infections [23]. Importantly, PLT-mediated parasite killing via secretion of PF4/CXCL4 has been described in several human-infective *Plasmodium* species [13, 24, 25]. However, the molecular basis of this apparent protective role of PLTs during Plasmodium infection requires further investigation.

Finally, a recent study reporting the metabolome of healthy PLTs when stimulated in vitro with *Pv*-IEs showed that PLTs undergo activation. However, the PLTs directly obtained from *P. vivax* patients have not been previously studied by molecular approaches due to the frequent thrombocytopenia that makes difficult to obtain these cells. Our goal in this study was to explore the role of PLTs during uncomplicated *P. vivax* malaria episodes, through proteome analysis of PLTs from *P. vivax* patients, the evaluation of key markers of PLTs and endothelial cell activation in plasma from patients and the in vitro effect of *Pv-*IEs on PLT activation as well as the effect of PLTs on parasite development.

## Methods

### Study population

Patients with *P. vivax* mono-infection confirmed by nested PCR, of any gender, and > 5 years old, were enrolled in two malaria-endemic locations: Quibdo (Choco, Colombia: latitude 5.6918802, longitude − 76.6583481) and Apartado (Uraba, Colombia: latitude 7.8829899, longitude − 76.6258698). A medical evaluation including epidemiological, as well as clinical information of the patients was recorded to guarantee the absence of severity criteria, according to the regulations previously stablished in Colombia [26].

A subsample of healthy volunteers with similar age to the group of patients (Mean ten years age difference) were enrolled in this study, providing they met the following eligibility criteria: no cardiovascular or chronic disease, no chronic consumption of anti-aggregate drugs, no fever-related symptoms during the last month, no malaria diagnosis during the last two months and malaria negative during enrollment, confirmed by thick blood film, rapid diagnostic test, and nested PCR.

### Diagnosis of *P. vivax* mono-infection

Detection of *P. vivax* in patients and healthy volunteers was performed by thick blood film as previously stablished [27]. Briefly, whole blood (4 mL) was collected with ethylenediaminetetraacetic acid (EDTA) to prepare thick blood films and to visualize the different stages of *P. vivax* parasites (rings, mature trophozoites, schizonts, and gametocytes) by Giemsa staining. Parasitemia was recorded using light microscopy to count the blood parasite density (parasites/µL). Only isolates with > 2000 asexual parasites/µL were used for in vitro studies as previously suggested [28].

*Plasmodium vivax* mono-infection was confirmed by nested polymerase chain reaction (PCR) as previously described [29]. Briefly, 200 µL EDTA-whole blood were collected on filter paper (Whatman #3) and dried at room temperature for DNA extraction, following the manufacturer instructions (Qiagen DNA minikit, Ref: 51,306). *Plasmodium* spp. genus was detected by universal PCR, using generic primers (0.250 µM) rPLU1 (5′- TCA AAG ATT AAG CCA TGC AAG TGA -3′) and rPLU5 (5′-CCT GTT GTT GCC TTA AAC TTC-3′), MgCl_2_ (2 mM), dNTPs (0.2 mM per nucleotide), Taq DNA Polymerase (2 U/µL) and 2 µL DNA. The universal PCR amplification was performed as follow: 1 cycle of 94° C × 4 min; 35 cycles of 94 °C × 30 seg, 55 °C × 1 min and 72 °C × 1 min and one cycle of final extension at 72 °C × 4 min. Then, *P. falciparum* or *P. vivax* species were detected by nested PCR using specific primers (0.250 µM) rFAL1–rFAL2 (*P. falciparum*) and rVIV1–rVIV2 (*P. vivax*), MgCl_2_ (2 mM), dNTPs (0.2 mM per nucleotide), Taq DNA Polymerase (2 U/µL) and 1µL of each product obtained by the universal PCR [29]. The nested PCR amplification was performed as follows: one cycle of 94° C × 4 min; 35 cycles of 94 °C × 30 seg, 58 °C × 1 min and 72 °C × 1 min and one cycle of final extension at 72 °C × 4 min. The amplified products were visualized by electrophoresis (Gel Red stained).

### PLT proteomes from patients with uncomplicated *P. vivax* malaria

For the PLT proteome study, a total of five patients with uncomplicated *P. vivax* malaria and five healthy controls with similar age (Mean ten years age difference) were enrolled. Whole citrated blood (30 mL) was collected from each participant and sent by airplane to the Malaria Reference Laboratory located in Medellín, within 4–6 h post collection to immediately extract the proteins.

PLT rich plasma (PRP) was obtained by centrifugating the whole blood at 100 g × 20 min. Then, PLT poor plasma (PPP) was obtained from a second centrifugation at 1200 *g* × 20 min. The PPP was frozen at − 80 °C for further analysis (described below). To purify PLTs, the PRP was mixed with HEPES buffer (1:1) and centrifuged at 200 *g* × 15 min to collect the supernatant. Then, PLTs were pelleted at 900 *g* × 15 min. PLTs were recovered and washed three times without suspension of the pellet, using washing buffer (sodium citrate 10 mM, 150 mM NaCl, 1 mM EDTA, and dextrose 1% at pH 7.4). All the centrifugation steps were done at room temperature to isolate PLTs from whole blood and prostaglandin I_2_ (PGI_2_ sodium, ref: BML-PG011-0010, Enzo life sciences) was added at 0.4 μM between each centrifugation step to avoid PLT activation.

The isolated PLTs from each sample were suspended in 200µL milliQ water containing Roche’s Complete™ Protease Inhibitor Cocktail (1×). Proteins were extracted by five cycles of freezing–thawing followed by centrifugation steps at 7000 *g* × 20 min (4 ºC). Total protein concentration was quantified by Bradford method and each sample was normalized to the sample with lowest protein concentration (Additional file 1: Fig. S1). The quality and integrity of extracted proteins were confirmed by 1D-electrophoresis (SDS-page) and Silver staining**.** The samples were stored at – 80 ºC until proteomic analysis at Glasgow Polyomics, University of Glasgow (United Kingdom).

At Glasgow Polyomics, protein samples were quantified and trypsin digested and quantified using the filter-aided sample preparation (FASP) protocol, as previously described [30]. A pooled internal standard (IS) was prepared with equal amounts of peptides from patients and healthy controls to normalize the data. Then, the peptides from samples and IS were tagged using 6-plex TMT® Mass Tagging Kit (Thermo Scientific). The LC–MS/MS was performed using an Orbitrap Elite MS (Thermo Scientific). Briefly, peptides were desalted and concentrated for 4 min on the trap column before being transferred to the analytical column using starting solvent conditions (5% solvent B). A water acetonitrile gradient was used; 5–45% v/v solvent B from 4 to 154 min, 45–100% v/v solvent B 154–154.1 min, held at 100% v/v solvent B 154.1–160 min and then re-equilibrated at starting conditions 5% solvent B for a total time of 165 min. A fixed solvent flow rate of 0.3 µl/min was used for the analytical column. The trap column solvent flowed at a fixed 25 µl/min using 1% acetonitrile with 0.05% formic acid. The Orbitrap Elite acquires a high-resolution precursor scan at 60,000 RP (over a mass range of m/z 380 – 1800) followed by collision-induced dissociation (CID) fragmentation and detection of the top 3 precursor ions from the MS scan in the linear ion trap. The three precursor ions are also subjected to Higher-energy collisional dissociation (HCD) in the HCD collision cell, followed by detection in the orbitrap. Singly charged ions were excluded from selection, while selected precursors were added to a dynamic exclusion list for 180 s.

Data analysis was performed using Proteome Discoverer (version 1.4), Excel 2010, and GraphPad Prism 5.0. Protein identification was performed using the Mascot search engine to interrogate the NCBI GenBank database, allowing a mass tolerance of 10 ppm for the precursor and 0.6 Dalton for MS/MS matching. Perseus software was used for statistical paired *t*-test analysis and a volcano plot was used to depict the protein abundances between Pv versus HC groups in LC–MS/MS, through the adjusted *p*-values (− log_10_) versus the fold change (Log_2_) obtained by *t*-test analysis. The significant differences between groups were defined by *p*-value < 0.05 for highly confident proteins with false discovery rate > 5% (FDR), Mascot score > 100, and coverage > 5%. The protein–protein interactions were explored with confidence > 0.9 using online database STRING (https://string-db.org/) which contains the records of human protein – protein characterization and interactions at theoretical as well as experimental levels. PLT protein functions were also searched using UNIPROT (https://www.uniprot.org) and platelet web databases (http://plateletweb.bioapps.biozentrum.uni-wuerzburg.de/plateletweb.php).

### PF4/CXCL4 and VWf levels in PPP from *P. vivax* malaria patients

PF4/CXCL4 levels were evaluated in PPP from each subject, using the Enzyme-linked Immunosorbent Assay (R&D Systems DY795), following manufacturer's instructions. Additionally, VWf levels were measured in citrated plasma at the Laboratorio Clinico Hematologico (Medellin, Colombia).

### In vitro PLT activation by *Pv*-IE parasites

#### Preparation of PLTs

A total of 10 mL of whole citrated blood was collected from healthy volunteers to obtain the PLT rich plasma (PRP) and purified PLTs as described above. Pelleted PLTs were suspended in RPMI medium (Sigma-Aldrich, R1640) without supplements and counted in Neubauer camera by light microscopy for further in vitro assays with *Pv*-IEs.

#### Collection and preparation of *Pv*-IEs

Whole blood (10 mL) was collected in heparin from patients with *P. vivax* mono-infection, having more than 2000 parasites/µL with > 60% of mature trophozoite stages [28, 31]. Samples were sent immediately to the Malaria Reference Laboratory for processing within 4–6 h from collection. Leukocytes were removed by centrifugation, and *Pv-*IE were washed using RPMI media without serum for further in vitro assays with PLTs.

To evaluate the PLT – *Pv-*IE interactions in vitro*,* the isolated *Pv-*IEs were concentrated at 50–100% parasitemia using percoll gradients at 45% as previously reported [31]. The concentrated *Pv-*IEs were suspended in RPMI medium at 5% parasitemia, and hematocrit 5% without serum supplement. Co-culture of *Pv*-IEs and PLTs (2.5 × 10^6^ PLT/mL counted in Neubauer camera) were incubated 60 min at 37 °C in N_2_ (90%), CO_2_ (5%), O_2_ (5%). Two negative controls were included: (1) *Pv*-IE cultured without PLTs, and (2) Uninfected erythrocytes (uEs) co-cultured with PLTs. Supernatants were collected from co-cultures and frozen at − 80 °C until analysis of PF4/CXCL4 levels by ELISA. All treatments were done in triplicate.

### Effect of PLTs on *Pv*-IE schizonts development

To evaluate the effect of PLTs on *Pv*-IE schizont development, *Pv-*IEs enriched with Percoll as described above, were suspended in RPMI media at parasitemia 5%, hematocrit 2%, and AB + serum 20% as previously reported [28]. *Pv-*IEs were stimulated with the following treatments: (1) healthy PLTs previously purified (2.5 × 10^6^ PLT/mL); (2) releasates of healthy PLTs (2.5 × 10^6^ PLT/mL) previously activated by collagen [10 µg/mL]; and (3) RPMI media as a control of parasite development to schizonts. All conditions were tested in triplicate.

The controls were monitored until schizont (replicating parasites ≥ three chromatins) formation between 20 and 24 h. Briefly, thick blood films from growth controls and treated wells were stained with Giemsa to determine the schizont frequency (%) by light microscopy in a total of 100 asexual parasites [28]. The frequency of gametocytes (sexual stage) was determined as a measure of parasite stress in culture. If ≥ 40% of schizonts were counted in growth controls, the isolate was considered successful for the analysis of PLT treatments. Schizont formation was compared between growth controls and treated parasites to determine the inhibition of schizont development.

## Results

### Clinical and epidemiological features of patients with *P. vivax* infection

A total of 48 patients with uncomplicated *P. vivax* malaria who came to the Hospital in Quibdó and Apartadó, were enrolled in this study between February–September 2018. All patients were diagnosed with *P. vivax* mono-infection by nested PCR and classified with uncomplicated *P. vivax* malaria as presented the following common symptoms: headache (79%), chills (79%), sweating (76%), adynamia (71%), myalgia (61%), arthralgia (58%), anorexia (58%), sickness (50%), low back pain (34%) and vomiting (32%).Twenty-six out of 48 (54.2%) patients were male. Additionally, a total of 25 healthy controls who met the eligibility criteria were recruited simultaneously in the same area where patients were enrolled and 13 out of 25 (52.0%) healthy controls were male.

General hematological parameters from patients and healthy controls, including blood PLT count, hemoglobin and hematocrit levels in both groups are shown in Table 1. As expected, thrombocytopenia (PLT count < 150.000/µL) was detected in 28 out of 48 (58%) patients, while all healthy controls showed normal PLT counts (150,000–450,000/µL).

**Table 1:** Characteristics of uncomplicated *P. vivax* malaria patients and healthy controls enrolled in the study

Complete blood cells count	PV* (n = 48)		HC* (n = 25)		*p*- value^a^
	Median	IQR	Median	IQR	
PLTs 10^3^/µL	133.0	95.5–166.3	246	203.0–277.5	** < 0.0001**
MPV (fL)	9.2	8.4–9.7	9.5	8.55–9.6	0.2753
PDW (Ratio)	18.1	16.9–19.5	16.8	16.2–17	** < 0.0001**
PCT (%)	0.1	0.08–0.14	0.218	0.192–0.264	** < 0.0001**
Hemoglobin (g/dL)	12.9	11.1–13.9	13.2	12.05–14.5	0.0972
Hematocrit (%)	38.4	33.5–42.3	39.7	35.9–43	0.1177
Age (years)	23	15–41	33	25–44	**0.0057**

*All subjects enrolled in the study include those from the proteomic cohort

*IQR* interquartile, *MPV* media PLT volume, *PDW* PLT distribution width, *PCT* plateletcrit, *PV* *P. vivax* patients. *HC* healthy controls

^a^Mann–Whitney U-test, significantly different to healthy controls (*p* < 0.05) shown in bold

### Changes in the PLT proteome from patients with *P. vivax* infection

A subgroup of five uncomplicated *P. vivax* malaria patients (male: 5/5; 100%) and five healthy volunteers (male: 3/5; 60%) were enrolled for PLT proteome assays. The supplemental information (Additional file 2: Table S1) contains relevant information of the subgroup of patients and healthy controls collected for proteomics assays.

**Fig. 1:**
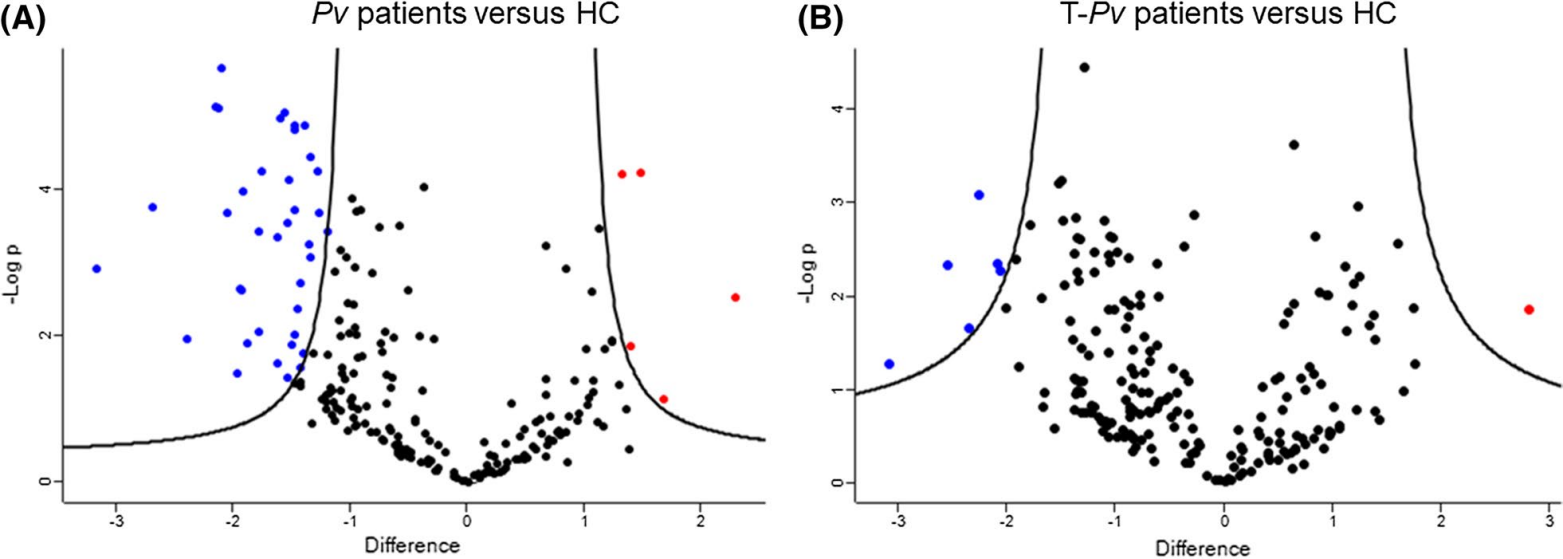
Differentially expressed PLT proteomes from uncomplicated *P. vivax* malaria patients. Volcano plots are shown from all shared protein entries and their abundance in the following conditions: **A** all patients with *P. vivax* infection versus healthy controls by a paired *t*-test analysis; **B** patients with < 100,000 PLTs/μL blood (thrombocytopenia) versus healthy controls by a paired *t*-test analysis. Each dot represents a protein mapped consistently to its –Log (*p*-value) on the ordinate axis and its difference (fold change) on the abscissa axis with FDR cutoff α (0.05). Red dots are increased proteins, blue dots are proteins decreased, and black dots are proteins equally expressed in all groups. The volcano plots were made using the Perseus Software platform

Through TMT-labelling approaches, a total of 215 protein entries were identified at FDR = 0.05% in patients and controls, using the SwissProt Human databank. The volcano plot depicted in Fig. 1 shows the proteins abundance in Pv versus HC groups by LC–MS/MS. A total of 38 PLT proteins were found to be decreased, and five proteins were increased in patients (Fig. 1). However, only 21 decreased, and five increased proteins were selected for further analysis because of the high-quality identification, defined as Mascot score > 100, FDR > 5%, > 2 peptides given match, and coverage > 5% (Table 2).

**Table 2:** PLT proteins differentially expressed in uncomplicated *P. vivax* malaria patients with thrombocytopenia

Accession number (Uniprot DB)	Gene	Protein	Coverage sequence	#Unique peptides	Mascot Score	All PV versus HC		T-PV versus HC*		Molecular function
						Fold change	*p*- Value^a^	Fold change	*p*- Value^a^	
P02776	CXCL4	Platelet factor 4/CXCL4	35.64	2	375.00	− 2.28	**0.003**	− 1.92	**0.0084**	Inhibits endothelial cell proliferation (E)
P60660	MYL6	Myosin light polypeptide 6	68.21	8	638.00	− 2.16	**0.001**	− 2.17	**0.001**	Regulatory light chain of myosin
O14950	MYL12B	Myosin regulatory light chain 12B	21.51	3	163.00	− 1.96	**0.0002**	− 1.92	**0.0025**	Regulate the cell contractile activity via its phosphorylation
O95810	CAVIN2	Caveolae-associated protein 2	17.88	7	597.00	− 1.73	**0.004**	− 1.50	**0.0171**	Plays a role in caveola formation
Q01518	CAP1	Adenylyl cyclase-assoc. protein 1	15.16	6	275.00	− 1.54	**0.0007**	− 1.41	**0.0044**	High specific activity and sensitivity to prostaglandins
P09493	TPM1	Tropomyosin alpha-1 chain	28.17	2	601.00	− 1.50	**0.016**	− 1.38	0.1335	Calcium-dependent regulation of cell contraction
P35579	MYH9	Myosin-9	44.85	74	4772.00	− 1.50	**0.007**	− 1.77	**0.0029**	Play a role in cytokinesis, cell shape, among others
P04406	GAPDH	Glyceraldehyde-3-phosphate dehydrogenase	49.25	12	972.00	− 1.47	**0.002**	− 1.44	**0.0069**	Delivery of nitric oxide to PLTs activated by thrombin
O75083	WDR1	WD repeat-containing protein 1	12.21	7	202.00	− 1.41	**0.0006**	− 1.26	** < 0.0001**	Decreased in PLTs activated by thrombin
P68032	ACTC1	Actin, alpha cardiac muscle 1	36.60	3	2649.00	− 1.41	**0.007**	− 1.06	**0.009**	Found in muscle tissues
P40197	GPV	Platelet glycoprotein 5	7.74	2.14	195.00	− 1.40	**0.025**	− 2.08	**0.0094**	Mediate PLTs adhesion to blood vessels through VWf
Q86UX7	FERMT3	Fermitin family homolog 3	21.44	10	1167.00	− 1.36	**0.0009**	− 1.20	**0.006**	Activate the integrin β [1–3] for adhesion to endothelial cells
P00338	LDHA	L-lactate dehydrogenase A chain	6.63	1	195.00	− 1.34	**0.003**	− 1.37	**0.0023**	Involved in cadherin and kinase binding
P61224	RAP1B	Ras-related protein Rap-1b	48.91	6	627.00	− 1.31	**0.015**	− 1.55	**0.0007**	Maintenance of endothelial polarity
P08567	PLEK	Pleckstrin	48.57	17	1688.00	− 1.30	**0.005**	− 1.29	**0.0123**	Regulates the fusion of granules to the membrane
A0A0A0MRZ8	IGKV3D-11	Immunoglobulin kappa variable 3D-11	7.83	1	114.00	− 1.30	0.534	− 2.54	**0.0097**	Region that participates in the antigen recognition
Q9H4B7	TUBB1	Tubulin beta-1 chain	20.40	6	602.00	− 1.24	**0.002**	− 1.05	**0.0025**	Main protein in microtubules. It binds GTP molecules
P11142	HSPA8	Heat shock cognate 71 kDa protein	17.18	9	488.00	− 1.24	**0.002**	− 1.18	0.0867	Protects the proteome from stress, folding and transport of newly synthesized polypeptides
P26038	MSN	Moesin	8.49	5	131.00	− 1.21	**0.014**	− 0.99	0.075	Involved in connections of cytoskeletal structures to the plasma membrane
P07737	PFN1	Profilin-1	79.29	14	2112.00	− 1.15	**0.003**	− 1.19	0.0516	Binds to actin affecting the structure of the cytoskeleton
Q92686	NRGN	Neurogranin	19.23	1	352.00	− 1.02	**0.019**	− 0.75	0.0583	Third messenger substrate during synaptic development and remodeling
P01009	SERPINA 1	alpha-1antitrypsin	35.89	16	136.00	1.33	**0.001**	1.22	**0.0136**	Thrombin formation and clotting by inhibition of APC system
P0DOX8	IGL1LC	Immunoglobulin lambda-1 light chain	40.74	3	753.00	1.49	**0.002**	− 1.60	**0.0051**	Antigen binding
P01011	SERPINA3	Alpha-1-antichymotrypsin	11.11	5	136.00	1.65	**0.048**	1.00	0.2071	PLTs degranulation
P01859	IGHG2	Immunoglobulin heavy constant gamma 2	52.45	8	1310.00	1.92	**0.001**	1.22	0.3589	Complement activation and phagocytosis
P0DJI8	SAA1	Serum amyloid A-1 protein	64.75	3	357.00	2.73	0.065	2.81	**0.0314**	Adhesion to PLTs modulating vascular endothelial adhesion

*T-Pv patients (Thrombocytopenic Pv patients) from the entire cohort of Pv patients

^a^Statistical significance was defined by *p*-value < 0.05 shown in bold. The table only depicts 26 proteins significantly different between patients and healthy controls. Also, those proteins were classified from lower to higher fold change

### Differentially expressed proteins in all-*Pv* patients

The proteins that were upregulated or downregulated in PLT proteomes from uncomplicated *P. vivax* malaria patients are depicted in Table 2 compared to healthy controls. Decreased proteins included PF4/CXCL4 and its variant1 PF4V1/CXCL4L1, and myosins (e.g., Myosin light polypeptide 6 (MYL6), myosin regulatory light chain 12B (MYL12B), tropomyosin alpha-1 chain (TPM1) and myosin-9 (MYH9), among others. On the other hand, the highly increased proteins included the acute phase reactants Serum Amyloid A-1 protein (SAA1), and SERPINAs 1 and 3, among others.

### Altered proteins in PLTs from thrombocytopenic *P. vivax* malaria patients

As shown in Additional file 2: Table S1, three out of five PLT proteomes came from patients with thrombocytopenia (PLTs < 100,000/µl) and the remaining 2 PLT proteomes were from patients with normal PLT count (150,000–450,000/µl). In the thrombocytopenic patients (T-*Pv*), a total of 3 proteins were identified as more strongly decreased compared to healthy controls. Remarkably, Glycoprotein V (GPV) and the Immunoglobulin kappa variable 3D-11 (IGKV3D-11) had twofold lower expression when compared to healthy controls (Table 2). In contrast, the PF4/CXCL4 was found to be strongly decreased in T-*Pv* patients compared to healthy controls while Serum Amyloid A1 (SAA1) was increased. The platelet proteomics of N-PV compared to T-PV patients did not show any statistically significant differences (data not shown), but further studies with increased patient numbers will be needed to investigate potential changes in platelets between these two groups.

### Biological significance of altered proteins and network of interactions

**Fig. 2:**
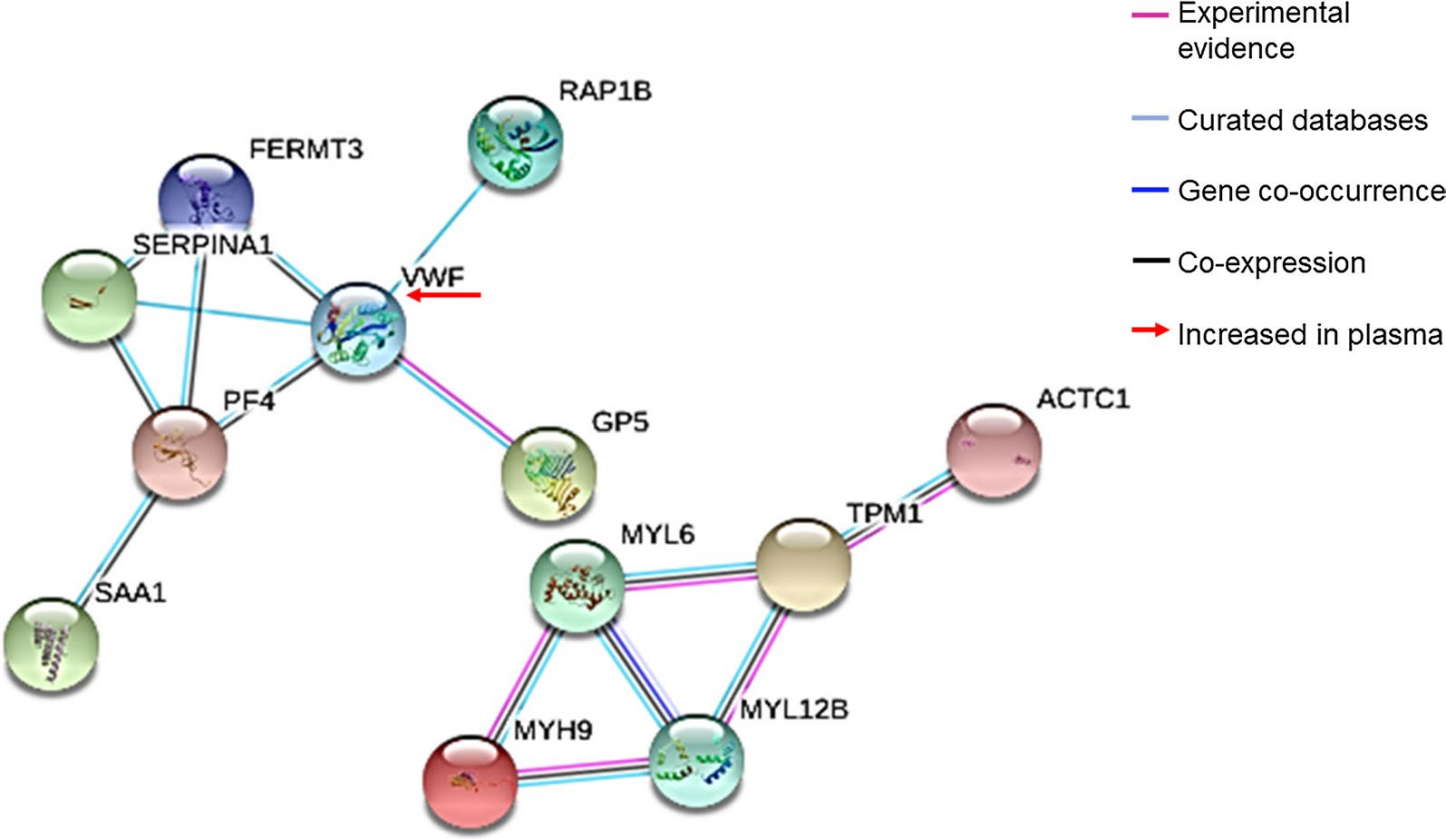
Proposed model of protein interactions regulating PLTs roles in the pathogenesis of *P. vivax* infection. The figure depicts the protein–protein significant interactions found by STRING analysis (*p*-value < 0.05) with a high level of confidence (Score > 0.9). Protein names: PF4/CXCL4 (platelet factor 4); SERPINA 1 (Alpha 1 Anti-trypsin); SSA1 (serum amyloid A 1 protein); FERMT3 (Fermitin 3); TPM1 (Tropomyosin 1); ACTC1 (Actin, alpha cardiac muscle 1); MYL12B (Myosin regulatory light chain 12B); MYL6 (Myosin light chain 6); MYH9 (Myosin heavy chain 9); GP5/V (Glycoprotein 5/V); RAP1B; VWf (Von Willebrand Factor). The lines depict the level of interaction evidence (Purple: experimental; gray: curated databases; blue: gene co-occurrence; black: co-expression). Even though VWf was not found differentially regulated in platelet proteomes, VWf plasma levels were higher in *P. vivax* patients (Fig. 3), thus it was included in the interactome to analyze its relationship with the most important differentially regulated PLT proteins (Red arrow). The black arrow depicts the protein GPV significantly decreased in the T-*Pv* group

The function(s) of the PLT proteins identified in this study were assigned based on UNIPROT and the PLT web database (http://plateletweb.bioapps.biozentrum.uni-wuerzburg.de/plateletweb.php) (Table 2). PLT expression and function of most of the identified proteins have been previously demonstrated at the experimental level. Most of these proteins have been previously found in the secretome, membrane, α-granules, or extracellular vesicles released from PLTs. Moreover, the decreased proteins identified in our study are related to the dynamic process of PLT shape change, adhesion, and activation through calcium mobilization into the cell and maintenance of the endothelial barrier (Fig. 2, Table 2).

A network of protein–protein interactions was built with all the differentially expressed proteins using the STRING web database. We also included the protein Von Willebrand Factor (VWf) in the network due to the substantial decrease of GPV in our proteomes, since it is well known that GPV is part of a protein complex (GPIb–GPIX–GPV) that interacts with VWf [41]. As shown in Fig. 2, a total of 12 interacting proteins in 15 significant edges (*p*-value = 0.0001) were found at a high level of confidence (> 90%), and seven interactions have previously been experimentally evidenced, mainly between myosins according to STTRING database. Further studies are needed to validate these molecular interactions during *P. vivax* infection and the progression of thrombocytopenia. Although a previous study reported that PLTs from malaria patients were highly activated [12], the relevance of PLT activation and blood coagulation in the pathophysiology of *P. vivax* malaria needs more investigation.

### PF4/CXCL4 and VWf levels in uncomplicated *P. vivax* infection

**Fig. 3:**
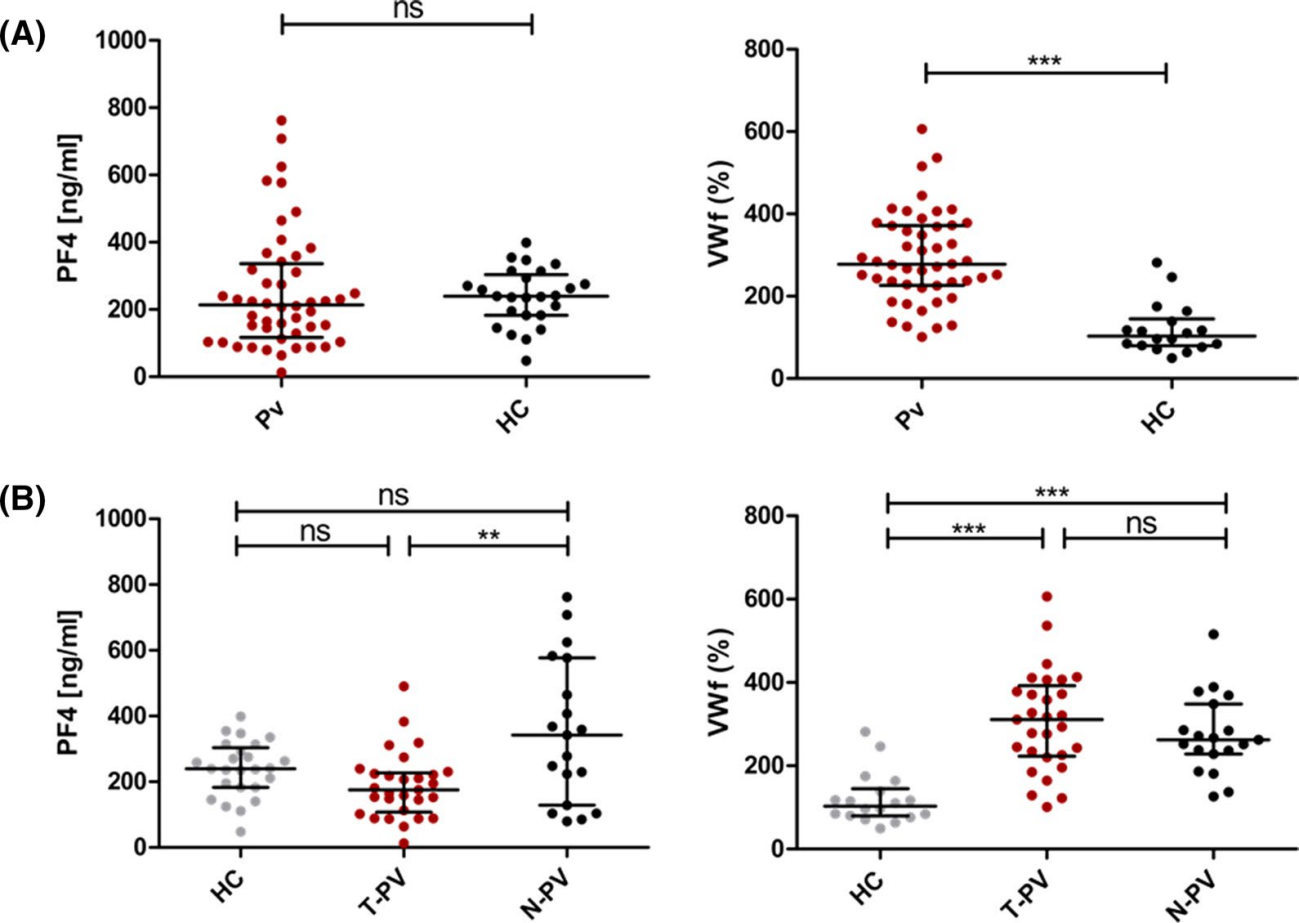
PF4/CXCL4 and VWf expression in plasma from uncomplicated *P. vivax* malaria patients. **A** Plasma concentration of PF4 and VWf from patients with *P. vivax* infection (PV) and Healthy controls (HC). **B** Plasma concentration of PF4 and VWf in patients with normal PLTs counts (N-*Pv*), patients with thrombocytopenia (T-*Pv*), and healthy controls (HC). All values are median (interquartile range) unless otherwise indicated. Mann–Whitney U-test, significantly different to control patients (*p < 0.05; **p < 0.01; ***p < 0.001)

To test whether the substantial decrease of PF4/CXCL4 in *P. vivax* PLTs could be due to PLT secretion, the plasma levels of this protein were measured. While no significant differences were detected between PV and HC groups (Fig. 3a), we found that PF4/CXCL4 plasma levels were significantly decreased in patients with low PLT count compared to patients with normal PLT count (Fig. 3b). Plasma levels of PF4/CXCL4 were not correlated with parasite density in any of these groups (data not shown: *r*^2^: − 0.18; *p* = 0.10). In contrast, the plasma levels of VWf were increased in all-*Pv* patients compared to HC (Fig. 3a and b).

### In vitro effect of *P. vivax* over PLTs—PF4/CXCL4 secretion

**Fig. 4:**
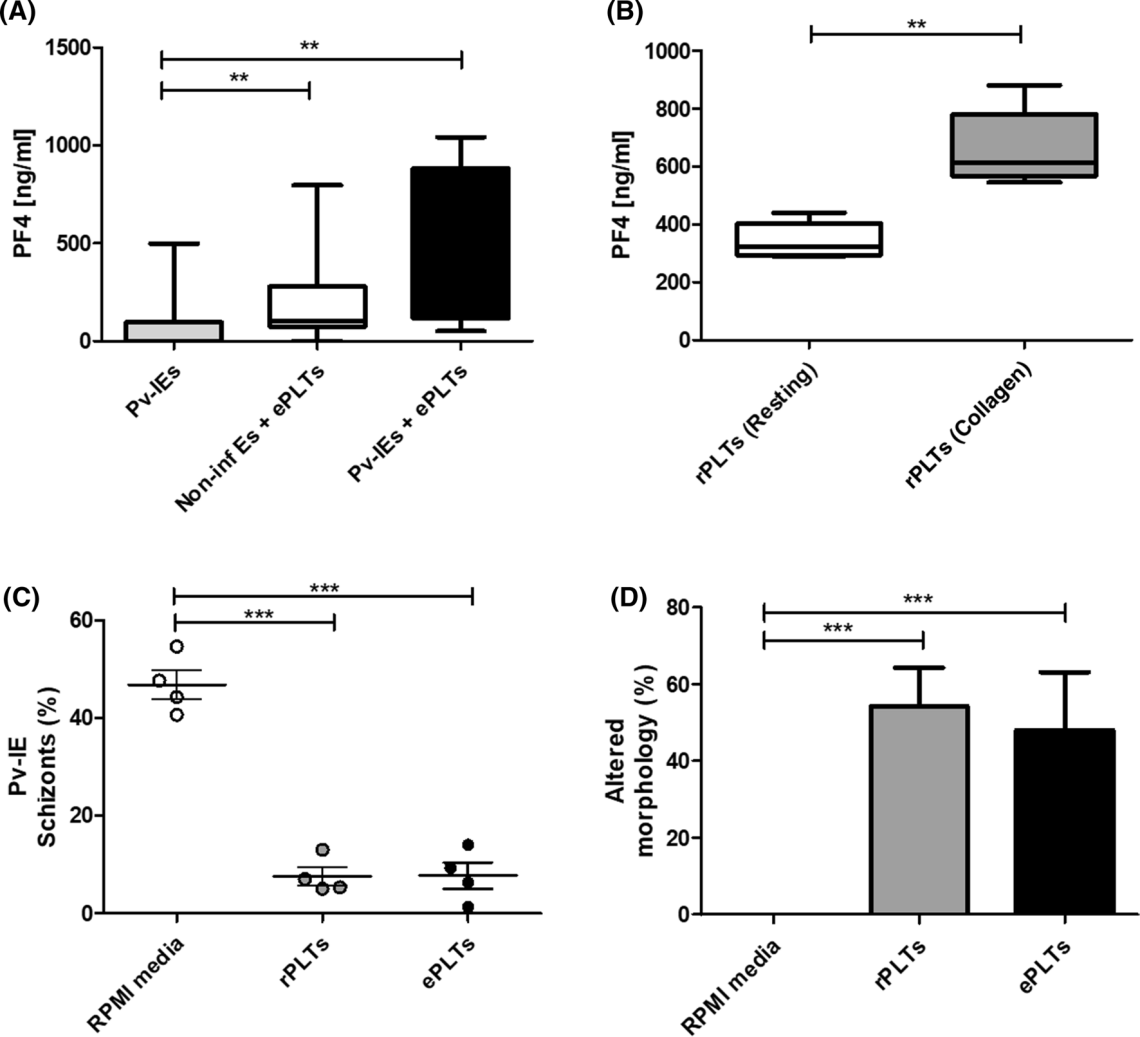
Platelet activation and parasite growth inhibition in vitro. **A** Platelet activation by *Pv*-IEs measured by PF4/CXCL4 release. Levels of PF4/CXCL4 from PLTs releasate (rPLTs) stimulated for 1 h at 37ºC with *Pv*-IE, uninfected erythrocytes (uEs), or RPMI media as a negative control. B Measurement of PF4 in PLTs releasate collected upon collagen stimulation. The rPLTs were obtained after stimulating PLTs with collagen (10 µg/mL) or PBS for 1 h at 37ºC, aliquoted and stored at -80ºC for further in vitro assays with fresh *P. vivax* isolates (see section C). This figure shows PF4/CXCL4 levels in five collagen-stimulated sPLT aliquots thawed at different times, to test PF4/CXCL4 stability in the frozen rPLTs. PF4/CXCL4 levels from sections A and B were measured by ELISA. **C** Effect of PLTs and PLTs releasate on *P.vivax* IE schizonts development. The figure depicts the effect of entire PLTs (ePLTs), releasate of PLTs activated by collagen 10 µg/mL (rPLTs), and RPMI media on *Pv-*IE schizont formation measured after 20–24 h of co-culturing with mature trophozoites. The frequency of schizont formation (%) was counted in Giemsa-stained thick blood films by light microscopy. **D** Frequency of *P.vivax* morphological alterations after culturing IEs with ePLTs or rPLTs. The frequency (%) of morphologically altered parasites was recorded in 100 *Pv*-IE. The morphological changes were defined as pycnotic nucleus, fragmented or condensed cytoplasm, and/or nucleus degradation. Kruskal–Wallis test have been shown statistical significance of mean and SD with *p*-value * < 0.05; **p < 0.01; ***p < 0.001

A total of 10 fresh *P. vivax* isolates with > 60% mature trophozoites (median of parasitemia 6800 parasites/µL; IQR: 5440–11,348) were used to test whether PLT activation, specifically the release of PF4/CXCL4, takes place in response to direct interaction with *Pv-*IEs. PF4/CXCL4 levels were higher in supernatants of PLTs co-cultured with *Pv-*IEs than those of PLTs with uninfected erythrocytes. These findings suggest that PLTs can indeed be activated and release granular content upon *Pv-*IEs stimulation (Fig. 4a).

### In vitro effect of PLTs over *P. vivax* schizonts development

To investigate whether entire PLTs (ePLTs) or PLT releasate compounds can impact *P. vivax* development, without the involvement of other vascular components in plasma, purified *Pv-*IEs were incubated in vitro with the following treatments: (1) resting ePLTs; or (2) releasates of collagen-activated PLTs. Four out of ten (40%) fresh *Pv*-IE isolates reached > 40% of schizonts (Fig. 4b). This percentage of successful maturation agrees with our previous study and is a common finding in *P. vivax* cultures [42]. Despite the small sample size of successful assays in which *Pv*-IEs developed to schizonts, we found a significant reduction of schizonts in *Pv-*IE co-cultured with ePLT and PLT releasate (Fig. 4c). Furthermore, after incubation with PLTs, characteristic features of dying *P. vivax* parasites were observed in Giemsa-stained samples, including the spread of parasite pigment (hemozoin) and pycnotic or parasite crisis forms (Fig. 4d). Also, an increase of gametocytes was observed that could be stress-related (data not shown).

## Discussion

Malaria illness is generally associated with periodic fever, chills, shivering, headache, nausea, vomiting, and many other clinical conditions [4]. In *P. vivax* malaria, several clinical conditions are due to the imbalance in pro- and anti-inflammatory cytokine production [23, 32]. In patients with acute *Plasmodium vivax* infection, the frequency of PLT counts under 150,000/µL can vary in different populations [33]. In this study, thrombocytopenia was present in 58% of patients, which agrees with previous reports in Colombia [5, 34].

Thrombocytopenia can be related to several variables during *P. vivax* infection, including parasitemia levels (> 20,000 parasites/µL are criteria of severity) [5], as well as host variables. In this study, we did not see differences in parasitemia levels nor in host variables such as age, gender, previous malaria episodes, or clinical outcomes in relation to PLTs count. Further studies in patients with a broad spectrum of Pv-related clinical conditions will be needed to address the relationship between PLT counts, parasite load, clinical outcomes, and disease severity.

In previous studies, PLTs from *P. vivax* patients have shown impaired aggregation when in vitro stimulated with agonists but the causes are unknown [10]. Our study analyzed the PLT proteome from patients with uncomplicated *P. vivax* infection to shed light on PLT functional pathways that might be activated. Remarkably, most proteins identified here were decreased in *P. vivax* patients. Of relevance, we found PF4/CXCL4, a small chemokine (7.8 kDa) released from α-granules during PLT activation, and with antimicrobial properties against bacteria, viruses, and parasites [17, 25, 35]. In malaria, circulating PLTs can act as a host defense, binding directly and killing intraerythrocytic parasites of four *Plasmodium* species infecting humans: *P. falciparum*, *P. vivax*, *P. malariae*, and *P. knowlesi* [25].

PLTs may have dual protective and pathogenic roles during *P. vivax* infection. PF4/CXCL4 has been shown to contribute with leukocyte trafficking into the injured cerebral vasculature during experimental cerebral malaria in mice [36]. Also, clinical evidence has demonstrated that plasma PF4/CXCL4 is a predictive biomarker of cerebral malaria in humans [37], while other studies suggested this protein is not important in malaria pathogenesis [25, 38]. Despite the decrease in PF4/CXCL4 found in our PLT proteomes from *P. vivax* patients, we did not detect increased plasma levels of this chemokine in our patients compared to healthy donors; however, high variability was observed within the malaria group. When comparing PF4 plasma levels between N-PV and T-PV patients, the latter showed lower levels of PF4. In combination with our in vitro results, our data supports the previously proposed model in which, upon PLT–*Pv*-IE interaction, locally released PF4/CXCL4 traffics into the digestive vacuole of *Plasmodium* parasites to induce death [24, 25]. Assuming an intracellular localization of PF4/CXCL4 could be related to the normal plasma levels in most of our patients, which is in line with a previous study reporting PLT–*Pv*-IE complexes and intraerythrocytic accumulation of this chemokine in uncomplicated *P. vivax* malaria patients, without detectable changes in plasma levels [25]. The higher amounts of plasma PF4/CXCL4 detected in few of our N-PV patients might be showing the early stages of PF4/CXCL4 response to the infection. In the case of T-PV patients, albeit not significantly different from healthy controls, the lower levels of PF4/CXCL4 in plasma could be the result of the decreased number of circulating platelets combined with the local secretion and internalization of the chemokine by *Pv*-IEs. Future studies will be needed to decipher the exact causes and mechanisms that lead to decreased amounts of intracellular PF4/CXCL4 cargo in platelets from PV patients; this could include not only platelet responses to IEs but also responses to other stimuli within the vasculature, and changes at the level of platelet production by megakaryocytes [39].

In *P. vivax* malaria, in vivo PLT–*Pv*-IE complexes have been previously observed [25]. However, the link between those complexes, the subsequent PLT activation, and a relationship to thrombocytopenia are unclear. To measure the consequences of PLT and *Pv*-IE interactions, we co-cultured healthy PLTs with *Pv*-IEs and showed that PLTs undergo activation releasing high levels of PF4/CXCL4 in response to *Pv*-IE stimulation. We also found that both intact PLTs and PLT releasates inhibit *P. vivax* schizonts formation causing phenotypic changes characteristic of dying parasites (namely spreading of parasite pigment suggestive of digestive vacuole dissolution). Our data agree with previous studies showing in vitro PLT killing of *P. falciparum* and *P. knowlesi* [13, 25, 40]. Importantly, these assays suggest that PLTs respond directly to the stimulus with *Pv*-IEs without the participation of other thrombotic or inflammatory signals.

Interestingly, our proteomic analysis also revealed decreased GPV in thrombocytopenic patients. This glycoprotein is a subunit of the GPIb-V-IX complex that constitutes the receptor for VWf and mediates the adhesion of PLTs to injured vascular surfaces (ref). Also, GPV can be cleaved from the surface of PLTs activated by thrombin, and ectodomain fragments of this glycoprotein may circulate in plasma, acting as potential thrombotic markers or modulators [41, 42]. We speculate that GPV may play an active role in the thrombocytopenia during uncomplicated *P. vivax* infection. However, it is currently unclear whether the decrease in GPV detected in our study is due to shedding from circulating PLTs or whether the megakaryocytes are producing PLTs with lower amounts of this glycoprotein. Our findings open questions on how the decrease in PLTs GPV relates to the higher levels of VWf found in plasma, and what is its relevance in the context of uncomplicated *P. vivax* malaria. Further studies are needed to elucidate the interactions between VWf and PLT receptors in *P. vivax* malaria, and the potential consequences to disease progression.

On the other hand, a few proteins were found increased in these proteomes. Serum Amyloid A 1 (SAA1) was the most upregulated protein with a 2.7-fold change. This is an acute-phase response protein synthesized and secreted by the liver in response to inflammatory cytokines, and it can reach up to 1000-fold increase in plasma [43]. Increased plasma levels of SAA1 have been previously reported during *P. vivax* infection [44]. Additionally, a significant increase of SAA1 protein has been found in severe *P. vivax* malaria patients through proteomic studies, suggesting it could be a potential predictive marker of disease progression [44, 45]. In our study, we suggest that SAA1 was increased in the proteomes probably due to PLT uptake from the serum, since PLTs have scavenging properties [46]. Further studies are required to determine the ability of PLTs to uptake and recycle SAA1 from the extracellular environment. Two important questions are whether SAA1 removal from the circulation could slow down the transition to severe malaria and whether PLTs can secrete stored SAA1 with physiological or pathological consequences in *P. vivax* malaria.

An inflammatory response in our *P. vivax* patients was evidenced by the elevated plasma levels of VWf, as well as increased abundance of SAA1 in PLTs. VWf is synthetized mainly by endothelial cells (ECs) but also by megakaryocytes and it is released from ECs and PLT α-granules upon cell activation [47]. Changes in plasma levels of VWf in malaria patients are associated with endothelial dysfunction and the risk of hemostasis dysregulation that could lead to intravascular coagulation and endothelial inflammation through increased formation of large VWf multimers and PLT aggregates [48, 49]. In this study, VWf was not found in the PLT proteomes of patients or healthy controls. We suggest this could be due to the low production of VWf from thrombopoietic cells (~ 15% of the total VWf) while the remaining 80–90% comes from endothelial cells [47]. The high levels of this protein found in plasma are also related to the endothelial activation previously evidenced in *P. vivax* malaria [50]. Furthermore, molecular studies are needed to fully clarify the interactions between VWf and PLTs at different stages of malaria disease.

The limitations of this study include the small sample size available for the proteomic analysis. One of the reasons was the challenge to select appropriate samples, keeping in mind that these were collected in a remote area with limited care facilities, and had to be shipped by airplane to the Malaria Reference Laboratory within 4–6 h post collection to avoid PLT activation. Another limitation of our study was the low amount of PLT proteins recovered from patient’s proteomes, ranging from 0.38 and 2.69 µg/µL (Additional file 1: Fig S1). This could be due to the low PLT count in blood (Additional file 2: Table S1) and/or to some degree of protein degradation during the extraction process. However, the internal standard prepared with all samples from patients and controls was used to normalize the data due to these limitations.

More studies are required to further characterize the implications of the changes we observed in the PLT proteome, and the interaction of PLTs with VWf through GPV in the vasculature, during uncomplicated *P. vivax* infection. It is important to complement our results with proteomic analysis from patients with several clinical outcomes, validating the findings through functional assays to understand the molecular pathways orchestrating PLT functions during the infection. In this sense, some challenges include the lack of good animal models, the difficulty in establishing in vitro cultures from *P. vivax* isolates, the inability to culture PLTs from patients, and the need to establish a culture of megakaryocytes to obtain PLTs in the laboratory for further in vitro studies.

## Conclusions

In summary, we show the first PLT proteome from patients with uncomplicated *P. vivax* malaria and thrombocytopenia as an effort to understand the role of PLTs in this disease. We have shown that PLT counts as well as PLT proteomes were altered in patients with uncomplicated *P. vivax* malaria. Endothelial activation takes place in uncomplicated *P. vivax* malaria as expected, which is supported by the increase of proinflammatory proteins (e.g., SAA1 in PLTs and VWf in plasma).

Interestingly, the decrease of intracellular PF4/CXCL4 and GPV suggest PLT activation and adhesion to the vascular endothelium, as well as an active role of PLTs in inhibiting schizont formation. Altogether, our findings suggest that during uncomplicated *P. vivax* infection, PLTs are active players interacting with both *Pv*-IEs and vascular components in the inflammatory process that takes place.

## Supplementary Information

**Additional file 1: Figure S1.** Integrity of PLT proteomes in samples. The figure depicts the band patterns detected by 1D SDS-PAGE and Silver staining in PLT protein samples from 5 patients with P. vivax infection and 5 healthy controls. MW: Molecular weight; kDa: Kilodaltons.

**Additional file 2: Table S1.** Main characteristics in the subgroup of patients and healthy controls enrolled for proteomic approaches

## Data Availability

The MS proteomics data in this paper will be deposited in the ProteomeX- change Consortium via the PRIDE partner repository.

## Abbreviations

PLTs: Platelets
Pv-IEs: *P. vivax* infected erythrocytes
Pf-IEs: *P. falciparum* infected erythrocytes
Pv: *P. vivax*
HC: Healthy controls
LC–MS: Liquid chromatography-Mass Spectrometry
ECs: Endothelial cells
EDTA: Ethylenediaminetetraacetic acid
PCR: Polymerase chain reaction
DNA: Deoxyrribunucleic acid
dNTP: Deoxynucleoside triphosphate
PRP: Platelet Rich Plasma
PPP: Platelet poor plasma
FDR: False discovery rate
MPV: Media PLT volume
PDW: PLT distribution width
PCT: Plateletcrit
PF4/CXCL4: Platelet Factor 4/CXCL4
SERPINAs: Serine protease inhibitors
MYL6: Myosin light polypeptide 6
MYL12B: Myosin regulatory light chain 12B
CAVIN2: Caveolae-associated protein 2
CAP1: Adenylyl cyclase-assoc. protein 1
TPM1: Tropomyosin alpha-1 chain
MYH9: Myosin-9
GAPDH: Glyceraldehyde-3-phosphate dehydrogenase
WDR1: WD repeat-containing protein 1
ACTC1: Actin, alpha cardiac muscle 1
GPV: Platelet glycoprotein 5
FERMT3: Fermitin family homolog 3
LDHA: L-lactate dehydrogenase A chain
RAPB1: Ras-related protein Rap-1b
PLEK: Pleckstrin
IGKV3D-11: Immunoglobulin kappa variable 3D-11
TUBB1: Tubulin beta-1 chain
HSPa8: Heat shock cognate 71 kDa protein
MSN: Moesin
PFN1: Profilin-1
NRGN: Neurogranin
SERPINA1: Alpha-1antitrypsin
IGL1LC: Immunoglobulin lambda-1 light chain
SERPINA3: Alpha-1-antichymotrypsin
IGHG2: Immunoglobulin heavy constant gamma 2
SAA1: Serum Amyloid A1
