# Supplementary figures and images for: Uncomplicated Plasmodium vivax malaria: mapping the proteome from circulating platelets
Source: Clin Proteomics. 2022 Jan 5;19:1. doi: 10.1186/s12014-021-09337-7 (PMC8903537; doi:10.1186/s12014-021-09337-7)

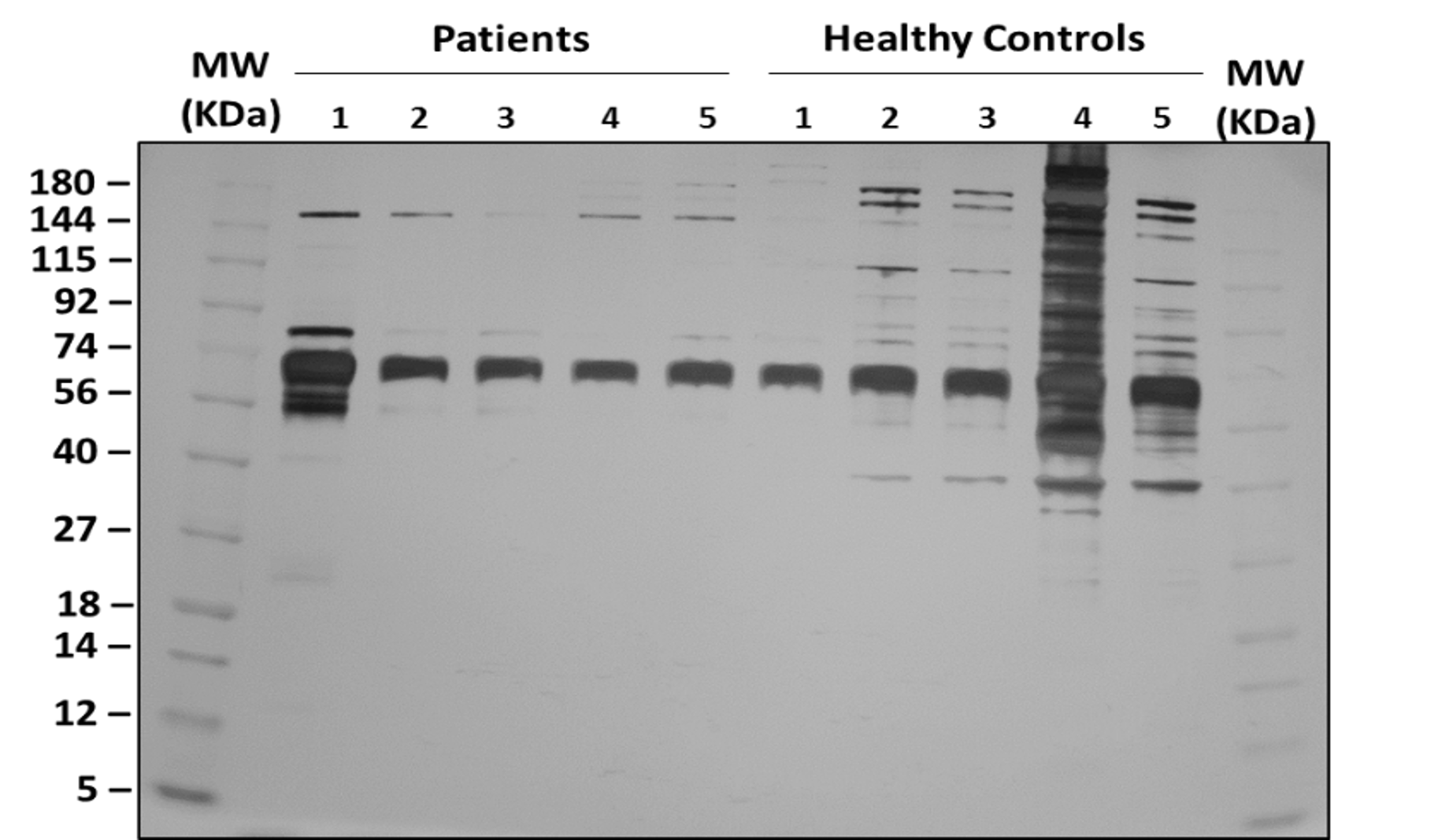

Supplement: Supplementary file 1 — Additional file 1: Figure S1. Integrity of PLT proteomes in samples. The figure depicts the band patterns detected by 1D SDS-PAGE and Silver staining in PLT protein samples from 5 patients with P. vivax infection and 5 healthy controls. MW: Molecular weight; kDa: Kilodaltons. [file 12014_2021_9337_MOESM1_ESM.tif]
