# Supplementary material for: Uncomplicated Plasmodium vivax malaria: mapping the proteome from circulating platelets
Source: Clin Proteomics. 2022 Jan 5;19:1. doi: 10.1186/s12014-021-09337-7 (PMC8903537; doi:10.1186/s12014-021-09337-7)
Supplement: Supplementary file 2 — Additional file 2: Table S1. Main characteristics in the subgroup of patients and healthy controls enrolled for proteomic approaches [file 12014_2021_9337_MOESM2_ESM.docx]

**Table S1. Main characteristics in the subgroup of patients and healthy controls enrolled for proteomic approaches**

|  | PV1 | HC1 | PV2 | HC2 | PV3 | HC3 | PV4 | HC4 | PV5 | HC5 |
| --- | --- | --- | --- | --- | --- | --- | --- | --- | --- | --- |
| Gender | M | M | M | M | M | F | M | F | M | M |
| Age (years) | 18 | 40 | 10 | 9 | 43 | 53 | 16 | 35 | 41 | 39 |
| Parasites/µL | 3,080 | -- | 6,880 | -- | 7,110 | -- | 6,220 | -- | 8,930 | -- |
| HGB [g/dL] | 13.2 | 13.9 | 13.3 | 13.8 | 12.9 | 14.5 | 13.7 | 11.9 | 12.9 | 14.5 |
| PLTs 10^3^/µL | 66 | 263 | 156 | 408 | 70 | 328 | 134 | 284 | 57 | 228 |
| VWF% | 244.8 | 182.2 | 262.5 | 62.5 | 372.3 | 114.5 | 164.1 | 80.7 | 100.7 | 40.4 |
| PF4 [ng/mL] | 319 | 182.6 | 707.8 | 211.1 | 112.7 | 140.3 | 181.2 | 111.6 | 382.6 | 236.5 |

**Legend Table S1.** Each pair of columns of PV and HC shows participants enrolled the same day in the study. PV: *P. vivax* patients (1 – 5). HC: Healthy controls (1 – 5). Gender: Male (M) or Female (F). HG: Hemoglobin. PLTs: Platelets. VWF%: Von Willebrand Factor (%). PF4: Platelet factor 4.
